# Orthodontists’ preference on type of rigid fixed functional appliance for skeletal Class II correction: A survey study

**DOI:** 10.4317/jced.57461

**Published:** 2020-10-01

**Authors:** Sarah Borghei, James Broadbent, Richard Stevens, Kishore Chaudhry, Karthikeyan Subramani

**Affiliations:** 1 Roseman University of Health Sciences, College of Dental Medicine, Henderson, NV, USA

## Abstract

**Background:**

Rigid fixed functional appliances are most commonly used to correct skeletal Class II malocclusions. The objective of this study was to assess orthodontists’ preference of different rigid fixed functional appliances used in the U.S.A for correction of skeletal Class II malocclusions.

**Material and Methods:**

A survey on use and preference of rigid fixed functional appliances for skeletal Class II correction was emailed to 2,227 members of the American Association of Orthodontists (AAO) in the U.S.A. Frequency distribution of different responses and their association with demographic factors was assessed.

**Results:**

Out of 140 orthodontists completing the survey, 110 responded as using rigid fixed functional appliances. Eight incomplete responses were eliminated from data analysis. 51.5% (68/132) orthodontists used rigid fixed functional appliances. The most preferred rigid fixed functional appliance was the Herbst appliance with 72% response followed by Mandibular Anterior Repositioning Appliance (24%) and AdvanSync (4%). There was no statistically significant difference in use of rigid fixed functional appliances between different age groups (*p*=0.284). However, the 40-54 age group used the most rigid fixed functional appliances in practice, followed by the 25-39 year age group and the 55-69 age group using these appliances the least. There was statistical significance between the type of practice setting one works in and the use of rigid fixed functional appliances in practice (*p*=0.022).

**Conclusions:**

About 52% of orthodontists use rigid fixed functional appliances to correct skeletal Class II malocclusions. The Herbst appliance is the most commonly used and most preferred amongst all rigid fixed functional appliances with a 72% preferred rate.

** Key words:**Orthodontic, Rigid fixed functional appliance, Skeletal Class II, Class II Malocclusion, Mandibular retrognathism, Herbst, Mandibular Anterior Repositioning Appliance (MARA), AdvanSync, Molar to molar, M2M.

## Introduction

The etiology for skeletal Class II malocclusion can be maxillary prognathism, mandibular retrognathism, or a combination of both (1). The most consistent diagnostic finding in skeletal Class II malocclusions is mandibular retrognathism (2,3). Functional appliances are used for treatment of skeletal Class II malocclusions caused due to mandibular retrusion and have been shown to produce a combination of dental and skeletal effects during the treatment to effectively reduce overjet in growing patients and normalize maxillary and mandibular positions in the anteroposterior plane (4,5). These appliances influence the jaws via the following mechanisms: remodeling of the mandibular condyle, remodeling of the glenoid fossa, repositioning the mandibular condyle in the glenoid fossa, and autorotation of the mandible. Most commonly, functional appliances are used to hold the mandible in a protrusive position to effectively correct Class II skeletal and dental relationships.

There are two types of functional appliances: removable and fixed. Ritto & Ferreira’s classification, which groups functional appliances according to the force systems the appliances use to move the mandible forward, consists of 3 types: flexible, rigid or hybrid (6). Some currently available rigid fixed functional orthopedic appliances include the Herbst appliance, AdvanSync (also known as Molar to Molar, M2M appliance; Ormco, USA), and Mandibular Anterior Repositioning Appliance (MARA) (4). These rigid fixed functional appliances have very similar mechanisms, in which they advance the mandible forward 24 hours a day, providing more stimulus for condylar remodeling and growth (7).

The original banded Herbst appliance was developed by Dr. Emil Herbst in 1909 and was reintroduced by Dr. Hans Pancherz in the late 1970’s. The Herbst appliance uses a bilateral telescopic mechanism consisting of a push rod and tube. It aims at keeping the mandible in an advanced position when the patient bites down (8). The AdvanSync (M2M) is a minor variation of the Herbst design, developed by Drs. Terry and Bill Dischinger (9). The M2M attaches the telescoping mechanism only to the maxillary and mandibular first permanent molar teeth. This directs the force only on the molars which has little to no protrusive force on the mandibular dentition, much like the Herbst appliance. Since the cantilever arm is shorter and smaller, orthodontics can be simultaneously completed while Class II correction is taking place, which reduces treatment time (10). In the early 1990s, Dr. Douglas Toll and Dr. Jim Eckhart developed the MARA as a more durable and less bulky alternative to the Herbst appliance, but with the same orthopedic and orthodontic correction of the skeletal Class II relationship (11). The patient is guided by the MARA appliance to habitually hold the mandible in an anterior position when the patient bites down (11,12).

With the variety of rigid fixed functional appliances available, there is a lack of information in the literature about the most preferred appliance for Class II correction. The purpose of this survey study was to evaluate the most preferred and commonly used type of rigid fixed functional appliance by orthodontists in the United States, to correct skeletal Class II malocclusion caused by mandibular retrognathism and explore why orthodontists prefer one appliance over the other.

## Material and Methods

A survey questionnaire was developed with an online survey platform (www.qualtrics.com). This study was approved by the Roseman University of Health Sciences Institutional Review Board. The survey questionnaire was reviewed and approved by American Association of Orthodontists (AAO) Partners in Research. The web link to the survey and a cover letter explaining the objectives were distributed to a random sample of 2,227 active U.S. members of the AAO. A reminder email was sent after 30 days and the survey remained open for another 15 days. After 45 days the data was collected.

Data was analyzed with IBM® SPSS® version 25. Active U.S. orthodontists who are a member of AAO, including orthodontic faculty, were included in the analysis. Retired orthodontists and orthodontic resident members of the AAO were excluded from the study. Descriptive statistics and association between participant demographic characteristics and the use and preference of fixed functional appliances were assessed using chi-square testing.

## Results

A total of 140 active U.S. based orthodontists completed the survey. Eight responses were incomplete and therefore eliminated from data analysis. From the responding participants, 98 (74%) were male and 34 (26%) were female orthodontists. Most of the respondents (89%) were between the ages of 40-69 years with only 11% of participants being between the ages of 25-39 years. One hundred and ten (83.3%) respondents had no affiliations to an academic institution. Private practice was the work setting for 112 orthodontists, whereas eleven worked in a corporate practice setting and the other nine worked in an academic setting. About 92% (121) respondents were taught about functional jaw orthopedics during their orthodontic residency program. About 95% of the participants in the age group of 40-54 years and 87.9% of the participants in the age group of 55-69 years were taught about functional jaw orthopedics during their residency program (Table 1).

**Table 1:**
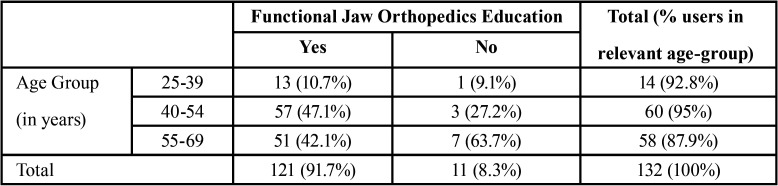
Functional jaw orthopedics education during residency program based on age group of orthodontists who participated in this survey.

Out of the 132 responses, there were 22 clinicians who did not use fixed functional appliances in their practice and were therefore excluded from fixed functional appliance related questions and analysis. When asked why they did not use fixed functional appliances, the highest response was that the fixed functional appliances are uncomforTable for the patient, followed by using fixed functional appliances was time consuming. Additionally, when asked if and how they addressed a skeletal Class II malocclusion caused by mandibular retrognathism, the responses varied between the use of Class II elastics, distalizers, camouflage or surgery.

**Figure 1:**
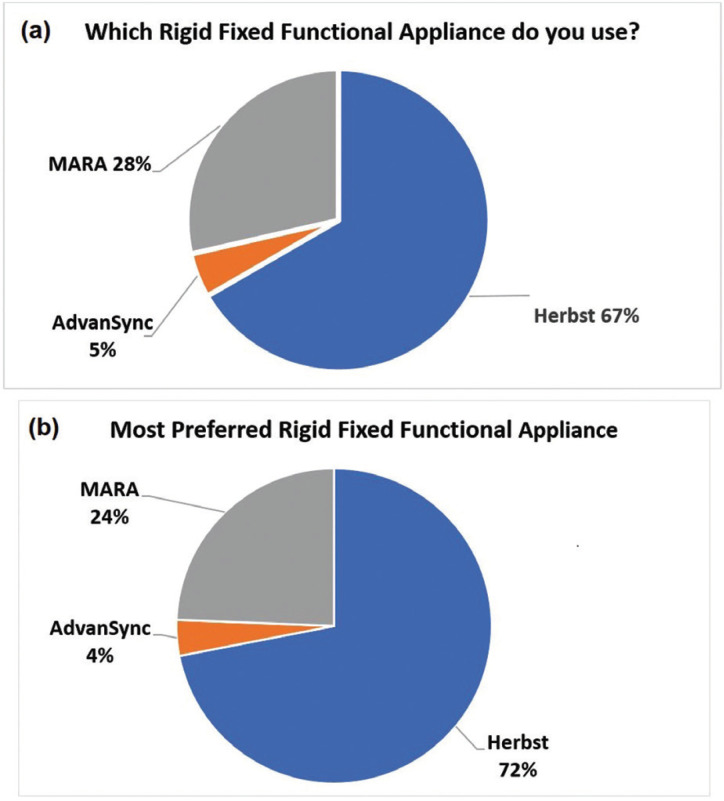
a. Pie chart showing the percentage of orthodontists using different rigid fixed functional appliances (Herbst/AdvanSync/MARA) in the U.S. b. Pie chart showing the percentage of most preferred rigid fixed functional appliances (Herbst/AdvanSync/MARA) in the U.S.

**Figure 2:**
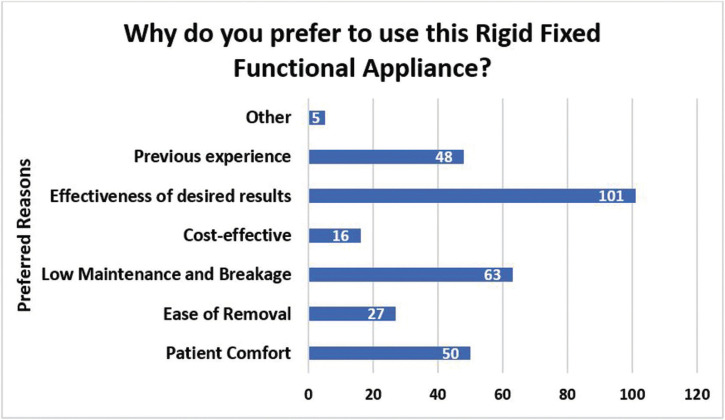
Graph depicting the most common reasons of an orthodontist’s preference to use a specific rigid fixed functional appliance*. *Respondents were instructed to select all applicable responses.

Amongst the remaining 110 (83.3%) respondents who indicated use of fixed functional appliances, 42 responses were determined as non-users since description of use related to other Class II dental correctors, distalizers, surgery or elastics. Thus, 51.5% (68/132) orthodontists use fixed functional appliances. Out of the 68 valid responses for those who used fixed functional appliances, 67% use the Herbst appliance, 28% use the MARA and 5% use the AdvanSync (Fig. 1a). Distribution was not statistically significant according to age group, gender, and whether or not they were taught about functional jaw orthopedics during residency (*p* = 0.284, *p* = 0.476, *p* = 0.481, respectively). There was an association between the type of practice setting where one worked in and the use of fixed functional appliances in practice (*p*= 0.022). Those who work in a private practice setting use fixed functional appliances more with 97 participants. The appliance that was most preferred from those who use fixed functional appliances was the Herbst appliance with a 72% preferential rate (Fig. 1b). The MARA had a 24% preferred rate and the AdvanSync was preferred amongst only 4% of participants who used fixed functional appliances in their practice. Out of those results, the Herbst appliance was favored by both male and female out of all appliances. When asked why they preferred that particular fixed functional appliance, the most common response was they achieved the desired results effectively (Fig. 2). The most desired outcome from using fixed functional appliances was to correct the dental malocclusion, followed by achieving mandibular advancement then facial balance.

**Figure 3:**
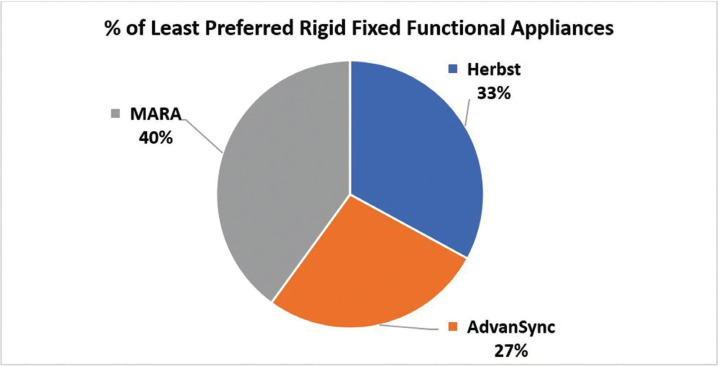
Pie chart depicting the percentage of least preferred rigid fixed functional appliances by the orthodontists who participated in this survey study.

When participants were asked which fixed functional appliance is their least favorite, the highest response was given to the MARA as the least favorite, followed by Herbst and lastly the AdvanSync (Fig. 3). Four responses were invalid and excluded since “other” was chosen with removable functional appliances as the response. 21 of the 118 participants who used fixed functional appliances did not have a least preferred type of fixed functional appliance and chose the “none” answer option. When asked why these appliances are their least favorite, the highest response for the MARA group was that the participant was unfamiliar/unacquainted with (unaware of) the appliance, followed by having a high rate of displacement and/or breakages and that they were uncomfortable for patients. The Herbst appliance was selected due to it being uncomfortable for patients. Those who least preferred the AdvanSync was due to their unfamiliarity with the appliance.

**Figure 4:**
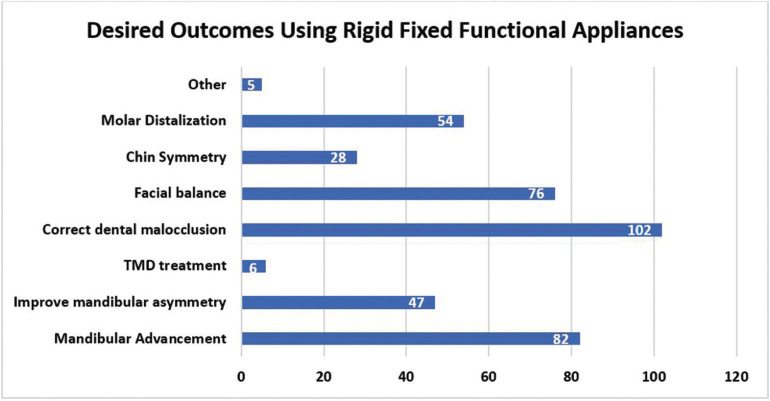
Bar graph depicting orthodontists’ desired outcomes using rigid fixed functional appliances*. *Respondents were instructed to select all applicable responses.

Figure 4 illustrates that the most desired outcomes and results in using these fixed appliances in general are that these appliances were effective in correcting dental malocclusion, advancing the mandible and achieving facial balance. When asked the question, “Approximately what percent of your Class II mandibular retrognathic cases in your practice are treated with fixed functional appliances?”, 29% responded with 0-19%, 26% use them for 20-39%, 7% use them 40-59% of the time, 19% use fixed functional appliances 60-79% of the time, 16% use them 80-99% and 3% of participants use fixed functional appliances 100% of the time to correct Class II malocclusions caused by mandibular retrognathism cases. Of the orthodontists who used fixed functional appliances in practice, 90 of them (82%) had the appliances fabricated by a lab and 20 of them (18%) had them fabricated in house.

## Discussion

Our study found that about half (51.5%) of orthodontists use some type of fixed functional appliance in practice. Within the surveyed participants, 88.3% of those in age group of 40-54 years use fixed functional appliances, followed by 85.7% of those in the 25-39 years group, and 77.6% of those in the 55-69 years use fixed functional appliances. The oldest age group, 55-69 years old, was likely to have completed residency in the late 1970’s to early 1980’s. During this time, Dr. Pancherz was rediscovering the Herbst appliance and its effect on mandibular advancement (8). Functional jaw orthopedics was not readily taught in all residencies since it was still being studied and observed in research. When asked if participants learned about functional jaw orthopedics during residency, 95% of those from the middle age range, 92.8% from the 25-39 age range, and 87.9% of the oldest age range were taught functional jaw orthopedics during residency. Despite the younger age group coming out of residency the most recently, not all were taught about functional jaw orthopedics. The middle age range showed the highest rate of using fixed functional appliances. This is likely because functional jaw orthopedics was introduced during the late 80’s and early 90’s, while the middle age group was in residency. This age range likely has more experience and exposure with these appliances than those who recently graduated.

Our study found that, of the orthodontists who use fixed functional appliances in clinical practice, majority prefer the Herbst appliance followed by the MARA and then the AdvanSync. Given the high response rate of functional jaw orthopedics education during residency and since the Herbst is one of the oldest fixed functional appliances, it is likely that orthodontist education and exposure of the Herbst appliance plays a role in the higher preference rate. The MARA and AdvanSync are newer appliances that were introduced in the 1990’s and 2000’s, respectively (10,11). Since the AdvanSync has only been around since the early 2000’s, this study found that, many respondents have never heard of, or used this appliance.

Table 2 shows the most preferred fixed functional appliance type divided by age group. Despite having all types of fixed functional appliances taught during residency, most practitioners in the youngest age group of 25-39 years still preferred the Herbst appliance. Within this age group, there was a high preference for Forsus and Powerscope. As these are not considered as fixed functional appliances, the responses were excluded from data analysis. While MARA was somewhat favored by the middle age group, Herbst was still the highest ranked between all age groups. According to the results, the Herbst appliance is still the most popularly used rigid fixed functional appliance.

**Table 2:**
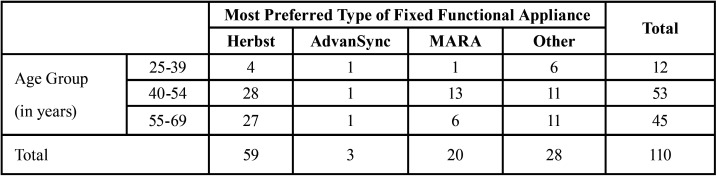
Table depicting the most preferred fixed functional appliance type by age groups of orthodontists who participated in this survey.

In our study, male orthodontists more frequently used fixed functional appliances. As of 2017, male orthodontists represent 72 percent of all professionally active orthodontists (13), which support why more males participated than females and why males have a higher rate of using rigid fixed functional appliances. The factors that were identified to have the most influence on practitioner’s choice and preference of fixed functional appliance were the following: effectiveness of desired results, low maintenance and breakage, and patient comfort (Fig. 2). Despite all appliances having similar results in correcting Class II skeletal malocclusions, the Herbst appliance was still the most preferred over the other appliances.

Interestingly enough, when asked which fixed functional appliance was their least favorite, MARA had the highest response rate of 40% followed by Herbst with a 33% response rate then AdvanSync last with 27% replying as least favorite. This was surprising given that when respondents had the opportunity to choose which fixed functional appliances they prefer, AdvanSync only had 4% of preference rate, while the MARA and Herbst had a preferred rate of 24% and 72%, respectively. This could be due to the survey respondent’s misunderstanding of the question. Participants may have thought their answers were limited to only the appliances they use or had previously selected in the survey. Had the question been framed differently, indicating appliance selection should be made independent of which appliances were previously selected in the survey or actually used by the respondent, results may have differed. Another possibility is that the orthodontist is restricted on which appliance they can use due to discretion of the practice owner, therefore they may not have all fixed functional appliance types available to them. When asked which fixed functional appliance is least preferred and why, the responses for AdvanSync indicated that many were unaware and have never heard of this appliance. The most likely reason is that there has been limited exposure to orthodontists in using the AdvanSync.

## Conclusions

• About 52% of orthodontists that completed this survey use rigid fixed functional appliances in their practice

• In this study, the Herbst appliance is the most commonly used and most preferred amongst all rigid fixed functional appliances with a 72% preferred rate.

• When asked why the Herbst appliance was the most preferred, many agreed that their desired results were effectively achieved.
